# Age-Associated Differences in MiRNA Signatures Are Restricted to CD45RO Negative T Cells and Are Associated with Changes in the Cellular Composition, Activation and Cellular Ageing

**DOI:** 10.1371/journal.pone.0137556

**Published:** 2015-09-11

**Authors:** Nato Teteloshvili, Joost Kluiver, Kornelis S. M. van der Geest, Roelof Jan van der Lei, Pytrick Jellema, Graham Pawelec, Elisabeth Brouwer, Bart-Jan Kroesen, Annemieke M. H. Boots, Anke van den Berg

**Affiliations:** 1 Department of Pathology and Medical Biology, University of Groningen, University Medical Center Groningen, Groningen, The Netherlands; 2 Department of Rheumatology and Clinical Immunology, University of Groningen, University Medical Center Groningen, Groningen, The Netherlands; 3 Department of Laboratory Medicine, University of Groningen, University Medical Center Groningen, Groningen, The Netherlands; 4 Groningen Research Initiative on Healthy Ageing and Immune Longevity (GRAIL), University of Groningen, University Medical Center Groningen, Groningen, The Netherlands; 5 Department of Internal Medicine II, Centre for Medical Research, University of Tübingen, Tübingen, Germany; 6 School of Science and Technology, Nottingham Trent University, Nottingham, United KIngdom; IPMC, CNRS UMR 7275 UNS, FRANCE

## Abstract

MicroRNAs (miRNAs) have emerged as important players in the regulation of T-cell functionality. However, comprehensive insight into the extent of age-related miRNA changes in T cells is lacking. We established miRNA expression patterns of CD45RO- naïve and CD45RO+ memory T-cell subsets isolated from peripheral blood cells from young and elderly individuals. Unsupervised clustering of the miRNA expression data revealed an age-related clustering in the CD45RO- T cells, while CD45RO+ T cells clustered based on expression of CD4 and CD8. Seventeen miRNAs showed an at least 2-fold up- or downregulation in CD45RO- T cells obtained from young as compared to old donors. Validation on the same and independent samples revealed a statistically significant age-related upregulation of miR-21, miR-223 and miR-15a. In a T-cell subset analysis focusing on known age-related phenotypic changes, we showed significantly higher miR-21 and miR-223 levels in CD8+CD45RO-CCR7- T_EMRA_ compared to CD45RO-CCR7+ T_NAIVE_-cells. Moreover, miR-21 but not miR-223 levels were significantly increased in CD45RO-CD31- post-thymic T_NAIVE_ cells as compared to thymic CD45RO-CD31+ T_NAIVE_ cells. Upon activation of CD45RO- T_NAIVE_ cells we observed a significant induction of miR-21 especially in CD4+ T cells, while miR-223 levels significantly decreased only in CD4+ T cells. Besides composition and activation-induced changes, we showed a borderline significant increase in miR-21 levels upon an increasing number of population doublings in CD4+ T-cell clones. Together, our results show that ageing related changes in miRNA expression are dominant in the CD45RO- T-cell compartment. The differential expression patterns can be explained by age related changes in T-cell composition, i.e. accumulation of CD8+ T_EMRA_ and CD4+ post-thymic expanded CD31- T cells and by cellular ageing, as demonstrated in a longitudinal clonal culture model.

## Introduction

Advanced age has been associated with defects of the immune system to mount appropriate antigen specific responses to pathogens. The most profound age-associated changes are observed in T cells. Due to thymus involution with age, the output of naïve T cells is reduced, while the proportion of memory T cells increases, thereby compromising the diversity of the T-cell pool.

Naïve T cells express CD45RA, while being negative for CD45RO [1]. Within the CD8+ T cell fraction, expression of CD45RA or CD45RO in combination with the C-C chemokine receptor type 7 (CCR7) is used to further define CCR7+CD45RA+(CD45RO-) naïve (T_NAIVE_), CCR7+CD45RA-(CD45RO+) central memory (T_CM_), CCR7-CD45RA-(CD45RO+) effector memory (T_EM_) and CCR7-CD45RA+(CD45RO)- late-stage effector memory (T_EMRA_) T-cell subsets [2]. Whether or not this model can also be applied to the CD4+ subset is still a matter of debate. Various age-related differences have been reported in the distribution of T-cell phenotypes in peripheral blood. For instance, the proportion of CD8+ T_EMRA_ cells is higher in elderly than in young individuals [3]. Within the CD4+CD45RO- T-cell population, the proportion of CD31- T_NAIVE_ cells increases with age, while the fraction of CD31+ T cells progressively decreases [4]. Kohler et al [5] characterized CD4+CD31+ T cells as recent thymic emigrants, while CD4+CD31- T cells represented central naïve peripherally expanded CD4+ T cells. Downregulation of surface expression of CD31 has been associated with homeostatic proliferation [6]. In elderly individuals, clonal expansion of memory T cells is required to preserve effective immune responses for combating antigenic re-challenges. This leads to a marked proliferative stress resulting in clonal exhaustion and senescence [7–9]. Human T-cell clones are characterized by altered cell surface and cytokine expression signatures that resemble the *in vivo* situation of chronic antigenic stress. Long-term cultured T-cell clones may thus represent a model for cellular ageing [10].

MiRNAs are a class of small non-coding RNAs that bind to mRNA transcripts of protein-coding genes in a sequence-specific manner. Based on the degree of sequence complementarity they induce degradation of the mRNA or repress translation [11]. A single miRNA potentially regulates up to several hundreds of target genes, thus orchestrating many pathways [12]. Differentiated cells in complex cellular systems are characterized by the expression of specific miRNA profiles. Moreover, miRNAs are fundamental to the regulation of complex cellular processes, such as those that regulate the immune system.

The contribution of changes in miRNA expression patterns to the age-associated decreased functionality of the immune system is largely unexplored. Differential miRNA expression patterns were shown in replicative *in vitro* and in *in vivo* models of CD8 T-cell ageing [13]. Some of the deregulated miRNAs were shown to be involved in the DNA damage response [14]. A role of miR-92a has been reported in age-related attrition of CD8+ T_NAIVE_ cells [15]. In addition, a role has been proposed for miR-181a in modulation of TCR sensitivity of CD4+ T cells upon ageing [16].

There are no comprehensive studies of differences in miRNA expression patterns in primary human T cells from younger and older donors. To address this issue, we analyzed miRNA expression in CD4+ and CD8+ T cells sorted based on expression of CD45RO from peripheral mononuclear blood cells (PBMCs) of young and elderly individuals. We observed age-related differences in miRNA expression predominantly within the CD45RO- T-cell compartment. These differences could be explained by a combination of age-associated accumulation of T_EMRA_ and CD31- T cells, T-cell activation and by cellular ageing. Finally, we assessed changes longitudinally in a clonal T cell culture model and found alterations in miRNA expression as a function of population doublings *in vitro*.

## Material and Methods

### Participants

In total 27 healthy young (≤ 30 yrs) and 24 healthy elderly (≥ 55 yrs) participants were included in this study. Demographic characteristics of all donors and miRNA analysis strategies are summarized in Table A in S1 File. Healthy young and old were recruited via advertisements and contacting the elderly association and were selected for healthiness based on the Senieur health admission criteria for immunogerontological studies [17]. None of the subjects had a history of infection, malignancy, autoimmune disease, chronic liver or kidney disease, alcohol or drug abuse, diabetes mellitus, current pregnancy or immunosuppressive therapy. An elevated blood pressure and the use of anti-hypertensive treatment were accepted.

T-cell clones were generated from phytohaemagglutinin (PHA)-stimulated peripheral blood mononuclear cells (PBMC) by limiting dilution in the presence of IL-2 using irradiated PBMCs as feeder cells [10,18]. In total, we included 16 CD4+ T-cell clones for each clone a sample with a low and a sample with a high number of population doublings. The T-cell clones were initially isolated from 5 individual donors (Table B in S1 File).

### Ethics statement

All participants provided written informed consent according to the Declaration of Helsinki to participate in this study that was approved by The Medical Ethical Committee (METC) (project number: 2009.118) of the University Medical Center Groningen UMCG.

### PBMC isolation and fluorescence-activated cell sorting (FACS) of human primary lymphocyte subsets

Peripheral blood was collected in heparin-containing vacutainer tubes (Becton Dickinson, Franklin Lakes, USA) and peripheral blood mononuclear cells (PBMC) were freshly isolated by density gradient centrifugation using Lymphoprep (Axis-Shield, Oslo, Norway) according to the manufacturer’s protocol. CD4 T cells were sorted by fluorescence-activated cell sorting (FACS) as CD3+CD4+ and CD8 T cells as CD3+CD4-. Within the CD4 and CD8 T-cell subsets, naïve (CD45RO-) and memory (CD45RO+), truly T_NAIVE_ (CD45RO-CCR7+) and terminally differentiated (T_EMRA_) cells (CD45RO-CCR7-); as well as CD3+ and CD31- populations were isolated using combinations of the following anti-human monoclonal antibodies: CD3-e450, CD4-A647 (eBioscience, Vienna, Austria), CD45RO-FITC, CCR7-PE, CCR7-PECY7 and CD31-PE (BD Bioscience, Breda, The Netherlands). See Figure A in in S1 File for sorting schemes.

### RNA extraction and purification

Total RNA was extracted using the miRNeasy Mini Kit (Qiagen, Venlo, The Netherlands) following the manufacturer’s instructions. Micro Bio-Spin^TM^ chromatography columns, supplied with Bio-Gel P-6 polyacrylamide gel matrices, were applied for efficient purification of RNA samples (Bio-Rad laboratories B.V. Veenendaal, The Netherlands). RNA concentration was measured on a NanoDrop ND-1000 Spectrophotometer (NanoDrop Technologies, Wilmington, USA). RNA samples with 260 / 280 and 260 / 230 ratio of ≥ 1.90 were used for further analysis.

### MiRNA profiling

Equal amounts of RNA samples from FACS sorted CD3+CD4+CD45RO+/- and CD3+CD4-CD45RO+/- T cells from healthy young (n = 5) and elderly (n = 5) donors were pooled for miRNA expression profiling. Microarray profiling was performed using the SurePrint Human miRNA Array Kit with 8x15K format (V2, sequence source from miRBase 10.1) from Agilent (G447013) according to manufacturer’s protocol (Agilent Technologies, Santa Clara, California, USA). Arrays were scanned using an Agilent scanner according to the manufacturer's instructions (Agilent Technologies). Array images were analyzed using Agilent feature extraction software. For data analysis, raw data were log_2_ transformed and normalized using a 90^th^ percentile shift using GeneSpring software (v.11.5.1). We excluded control probes and probes detecting viral miRNAs. MiRNAs detected in at least 7 out of 8 samples were included for further analysis. Visualization of normalized data and hierarchical clustering was performed with Pearson correlation distance and complete linkage clustering parameters using Genesis software (v.1.7.6) (Graz University of Technology, Graz, Austria) [19]. The microarray data were deposited in Gene Expression Omnibus and are accessible with the following series record number: GSE69191.

### Quantitative RT-PCR

MiRNA expression levels were determined by quantitative (q)RT-PCR. Multiplexed cDNA synthesis for up to 6 specific miRNAs was performed using Taqman MicroRNA Reverse transcription kit with a multiplexed reverse transcription primers of TaqMan microRNA Assays (Life Technologies, Carlsbad, USA): for miR-21 (000397), miR-223 (002295), miR-451 (001141), miR-22 (000398), miR-15a (000389), miR-197 (000497), miR-766 (001986), miR-574-3p (002349), miR-328 (000543), miR-483-3p (CSS07FR), miR-885-5p (241451_mat), miR-28-5p (000411) and RNU49 (001005) as described earlier [20]. RNU49 served as a reference gene to normalize miRNA expression levels.

All PCR reactions were run in triplicate. Mean cycle threshold (Ct) values were quantified with the Sequence Detection Software (SDS, version 2.3, Life Technologies, Amsterdam, The Netherlands). Relative expression levels were quantified using the 2^–ΔCt^ (Δ C_t_ = C_t_ gene—C_t_ reference gene).

### T cell stimulation with αCD3/αCD28 monoclonal antibodies (Abs)

Culture plates were coated with goat-anti-mouse IgG2a Ab (Cat. No. 1080–01, Southern Biotechnology, Uden, The Netherlands) and mouse anti-human CD3 hybridoma supernatant (clone WT32, IgG2a). CD3+CD4+CD45RO- and CD3+CD4-CD45RO- T cells were seeded at a density of 0,5x10^6^ cells/mL in RPMI medium (Lonza, Breda, The Netherlands) supplemented with 5% V/V mouse anti-human CD28 hybridoma (clone 20–4669, IgG1), 2% HSA (Sanquin, Amsterdam, The Netherlands). Cells were split and placed in fresh medium supplemented with αCD3/αCD28 on day 3, 5 and 7. On day 10 cells were harvested and stained for FACS analysis or lysed for RNA isolation.

### Analysis of cell surface markers

Cell surface markers on *in vitro* activated T cells were assessed using mAbs against human CD25-PE (BC96) (eBioscience, Vienna, Austria) and CD45RO-FITC (UCHL1) (BD Biosciences, Breda, The Netherlands). Cells were analyzed on BD LSR-II Flow Cytometer by Diva software (BD Biosciences). Data analysis was performed on Kaluza Flow Analysis Software (1.2) (Beckman Coulter, Woerden, The Netherlands).

### Statistical analysis

Results obtained from qRT-PCR are expressed as median respectively. Unpaired samples were compared using the Mann-Whitney test and paired samples using the Wilcoxon signed-rank test. For comparisons of paired unstimulated and stimulated CD4+ and CD8+ T cells, we applied the Wilcoxon matched pairs test. T-cell clones with low and high PD were compared using a paired Wilcoxon signed-rank test. Statistical analyses were performed with GraphPad Prism version 5.0 (GraphPad Software, San Diego, CA, USA) and SPSS Statistics version 22.0 (IBM Corp. Armonk, NY, USA). P < 0.05 was considered statistically significant.

## Results

### Age-related miRNA expression differences are restricted to CD45RO- T cells

**Fig 1:**
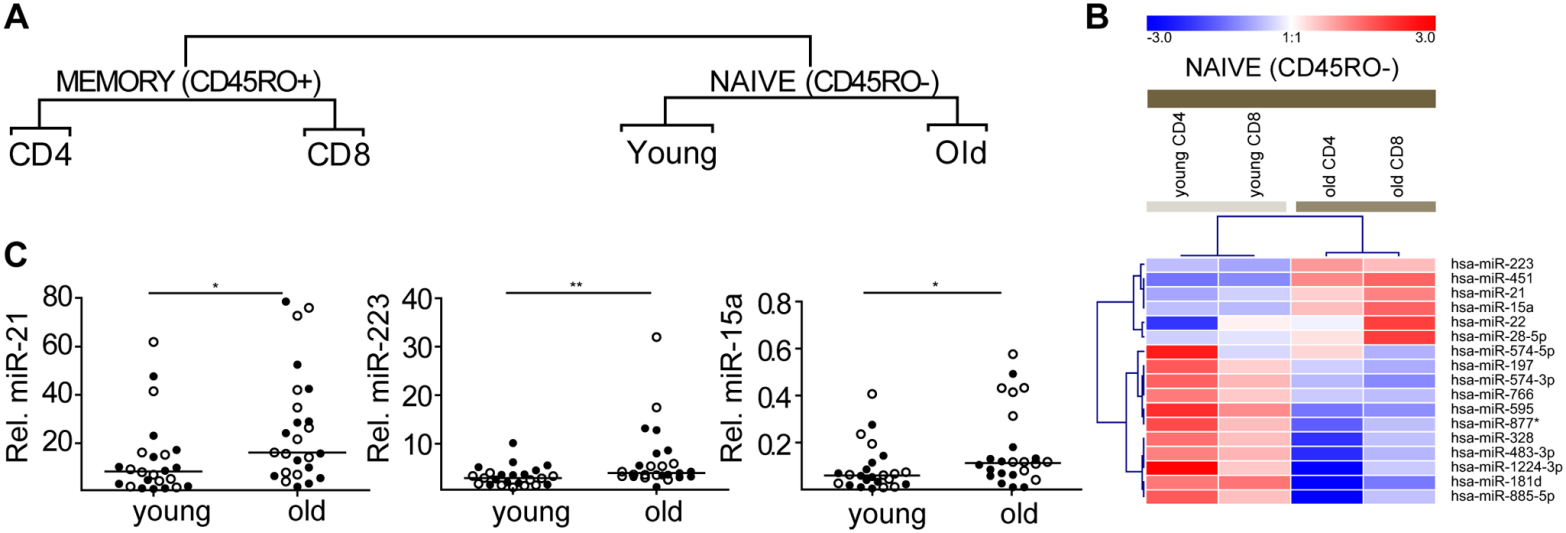
Age-related differences in miRNA expression are predominantly found in CD45RO- T cells. **(A)** Unsupervised hierarchical cluster analysis of 166 miRNAs revealed a clear distinction between CD45RO- and CD45RO+ T cells. Within the CD45RO- subgroup the next separation was based on age, whereas in CD45RO+ T cells the next clustering was based on expression of CD4/CD8 (the complete heatmap can be seen in Figure C in S1 File). **(B)** Heatmap of miRNAs differentially expressed in young versus old CD45RO- T cells (n = 17) using a 2-fold expression change cut-off (≥ 2 fold) between young and old CD45RO- T cells. The blue to red gradient on the heatmap indicates relative miRNA expression levels varying from low (blue) to high (red). **(C)** Relative expression levels in CD45RO- T cells as determined by qRT-PCR of miR-21, miR-223 and miR-15a in CD45RO- T cells from young and old donors. MiRNA expression levels were normalized to the expression levels of RNU49 (lines indicates median). Filled circles indicate CD4 T cells and open circles indicate CD8 T cells, *p ≤ 0.05, **p ≤ 0.01.

To identify age related differences in T cells, we compared miRNA expression profiles from sorted T cells from peripheral blood of 5 healthy young (median age 27) and 5 healthy old (median age 64) donors. CD4 and CD8 T cells were sorted from the CD45RO- and CD45RO+ gates (Figure A in S1 File). Purity of the T-cell subsets are shown in Figure B in S1 File. Equal amounts of RNA were pooled for each T-cell subset resulting in a total of 8 T-cell subset pools. MiRNA signatures were generated from these pools by miRNA expression arrays. Unsupervised hierarchical clustering of the 166 miRNAs that were detected in at least 7 out of 8 samples revealed a clustering into two main groups, i.e. CD45RO- and CD45RO+ T-cells (Table C and Figure C in S1 File). In the CD45RO- T cell subset, the clustering was based on age, irrespective of CD4 and CD8 expression status (Fig 1A). In the CD45RO+ T-cell subset, the clustering was between CD4+ and CD8+ T cells, irrespective of age. Based on this marked difference we focused our further analysis on the CD45RO- T-cell subset.

Seventeen miRNAs showed a ≥ 2-fold difference in expression level between young and old CD45RO- T cells (Fig 1B). Based on miRNA expression level and availability of qRT-PCR assays, 13 out of the 17 miRNAs were selected for validation on the individual samples of the pools used for the array analysis combined with a second independent cohort (young; n = 12, median age 27; old; n = 13, median age 65) (Table A and Table D in S1 File). Array results of miR-21, miR-223 and miR-15a were validated on the combined cohort (Fig 1C). MiR-28-5p expression levels were increased in old compared to young subjects, but this difference did not reach significance (Figure D in S1 File). Of the other selected miRNAs, we could not validate differences between young and old donors for miR-197, miR-766, miR-328 and miR-451 (Figure D in S1 File). We next studied if the observed changes could be assigned to ageing related changes in the T-cell composition. We focused these analyses on miR-21 and miR-223, as the miR-15a levels were very low.

### Increased numbers of CD8+ T_EMRA_ cells in elderly contributes to the higher miR-21 and miR-223 levels in CD45RO- T cells

**Fig 2:**
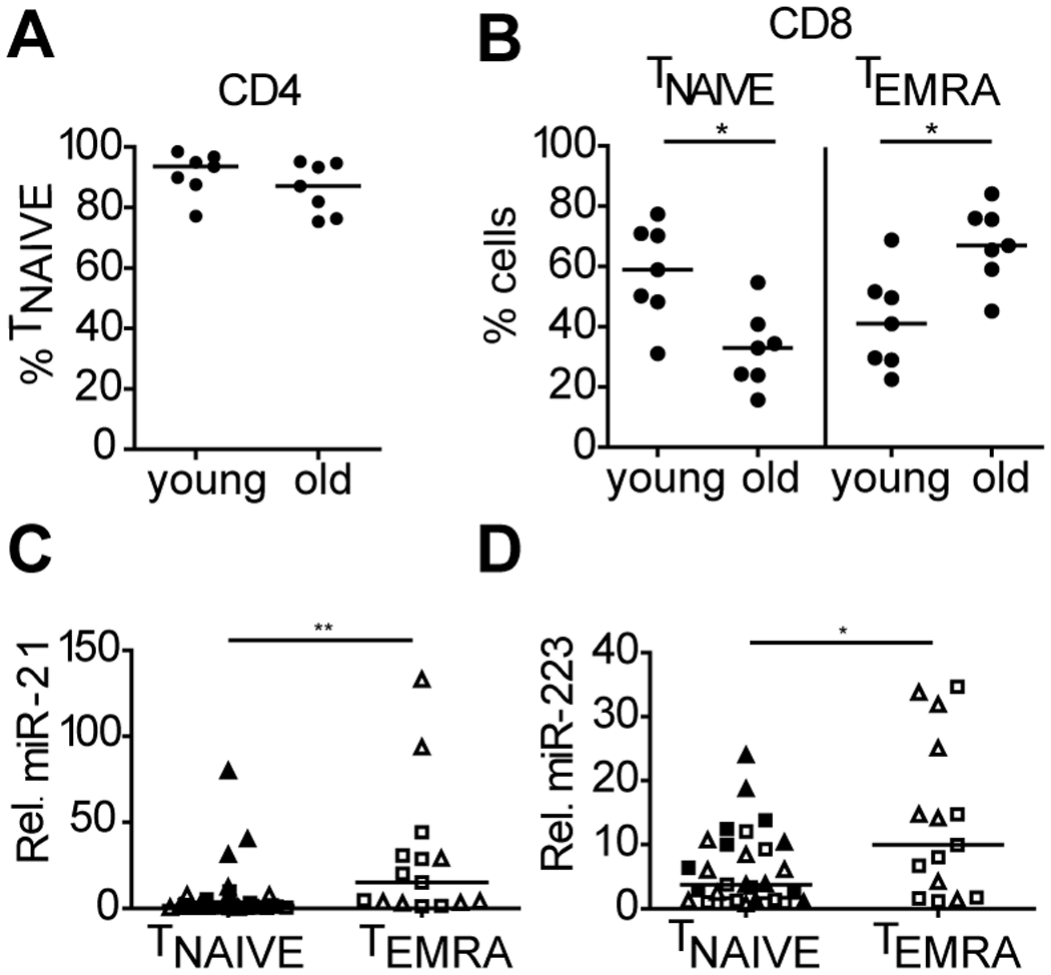
Age-related upregulation of miR-21 and miR-223 can be partly explained by the accumulation of T_EMRA_ (CD8 T cells) in the CD45RO- T-cell compartment. Percentage of **(A)** T_NAIVE_ CD4+CD45RO-CCR7+ and **(B)** T_ERMA_ CD4-CD45RO-CCR7- T cells in young and old donors. Relative expression of **(C)** miR-21 and **(D)** miR-223 in T_NAIVE_ (CD4 and CD8) and T_ERMA_ (CD8) T cells in young vs old donors. MiRNA expression was normalized to the expression of RNU49 (Lines indicate median). Squares indicate cells from young and triangles cells from elderly donors. Filled symbols indicate CD4+ T cells and open symbols indicate CD8+ T cells. *p ≤ 0.05, **p ≤ 0.01.

Loss of CCR7 expression is a hallmark of differentiation from central memory to effector and late effector CD8+ T_EMRA_ cells. To explore if the observed changes in miR-21 and miR-223 levels are related to the increase of T_EMRA_ cells in elderly, we sorted CD45RO-CCR7+ T_NAIVE_ and CD45RO-CCR7- T_EMRA_ cells from peripheral blood of 7 healthy young (median age 27) and 7 healthy old (median age 67) donors (Figure A in S1 File). Purity of the T-cell subsets are shown in Figure B in S1 File. The percentage of CD45RO-CCR7+ T_NAIVE_ cells within the CD4+ gate was high for both young (median is 93,6%) and old donors (median is 87,1%) (Fig 2A). The yield of CD4+CD45RO-CCR7- T_EMRA_ cells was insufficient for the qRT-PCR analysis. For CD8+ T cells we observed a significantly lower percentage of CD45RO-CCR7+ T_NAIVE_ cells in elderly donors (median of 33% in elderly vs 59% in young) with a concomitant significant increase in the percentage of T_EMRA_ cells (median percentage 67% in elderly vs 41% in young) (Fig 2B). QRT-PCR analysis revealed significant higher expression of both miR-21 and miR-223 in the T_EMRA_ populations when compared to T_NAIVE_ cells (Fig 2C and 2D). Hence, increased expression of both miRNAs in the CD45RO- T-cell subset upon ageing can at least in part be explained by the age-associated accumulation of CD8 T_EMRA_ cells.

### Increased numbers of CD31- central naïve T cells contributes to the increased levels of miR-21 in CD45RO- T cells

**Fig 3:**
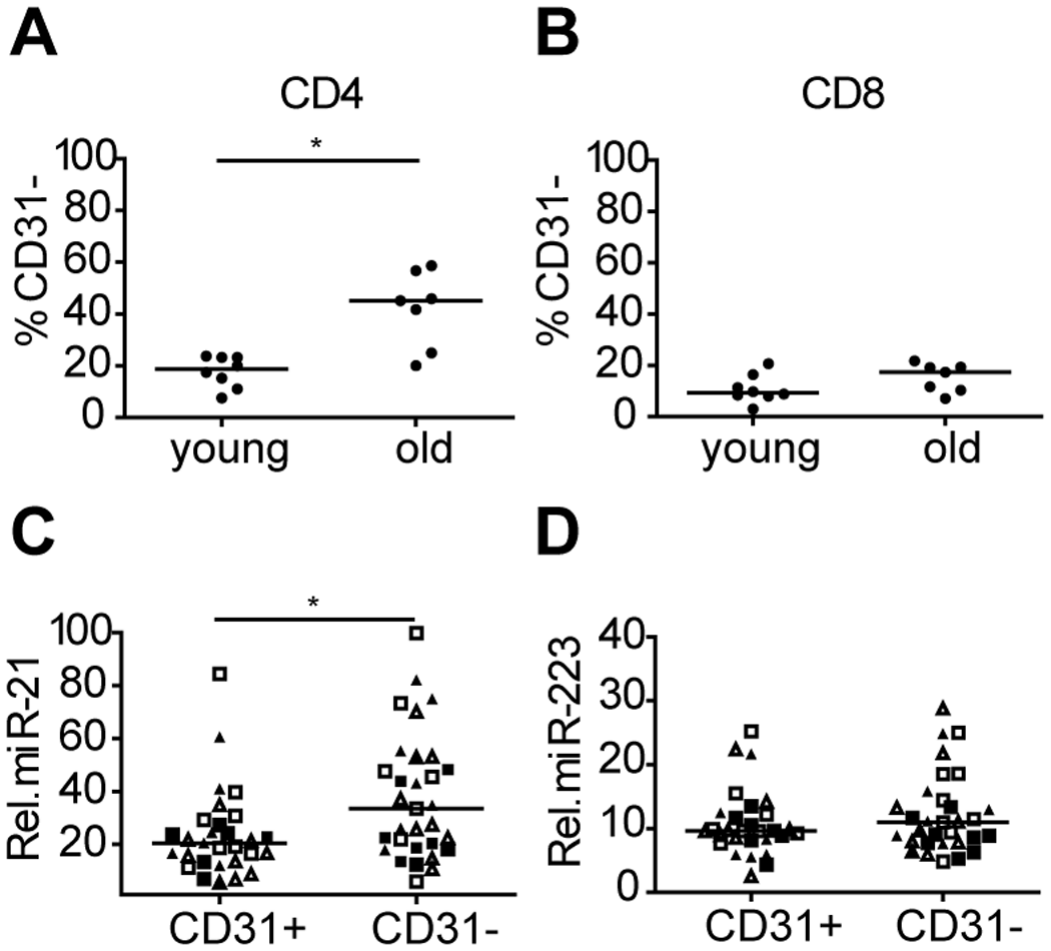
MiR-21 is upregulated in CD31- T cells in the CD45RO- compartment. Percentage of CD31+ and CD31- in **(A)** CD4+CD45RO- and **(B)** CD4-CD45RO- T cells in young and old donors. Relative expression of **(C)** miR-21 and **(D)** miR-223 in CD31-and CD31+ T-cell subset sorted from the CD45RO- T-cell compartment. MiRNA expression was normalized to the expression of RNU49 (line indicates median). Squares indicate cells from young and triangles indicate cells from old donors. Filled symbols indicate CD4+ T cells and open symbols indicate CD8+ T cells. *p ≤ 0.05.

As a next step, we studied expression patterns of miR-21 and miR-223 in relation to CD31 expression as it is known that CD31- central naïve T cells increase with age especially in the CD4+ T cell subset. CD31+ and CD31- T cells were sorted from the CD45RO- compartment for both CD4 and CD8 from PBMCs of 8 healthy young (median age 28) and 7 healthy old (median age 75) donors (Figure A in S1 File). Purity of the T-cell subsets are given in Figure B in S1 File. The percentage of CD4+CD31- T cells was significantly higher in elderly donors (median is 45%) when compared to younger individuals (median is 19%). In the CD8 compartment, we observed a slightly higher, albeit not significant, percentage of CD31- T cells in elderly (median is 17%) compared to young donors (median is 9,3%)(Fig 3A and 3B). The expression level of miR-21 was significantly increased in CD45RO-CD31- T cells compared to CD45RO-CD31+ T cells, whereas the miR-223 levels were similar in CD31+ and CD31- T cells (Fig 3C and 3D). Thus, the accumulation of CD31- T cells contributes to the observed age related increase of miR-21 levels, especially in the CD4+CD45RO- T cell compartment in which CD31 loss is most pronounced.

### MiR-21 is upregulated upon activation of CD45RO- T_NAIVE_ cells

**Fig 4:**
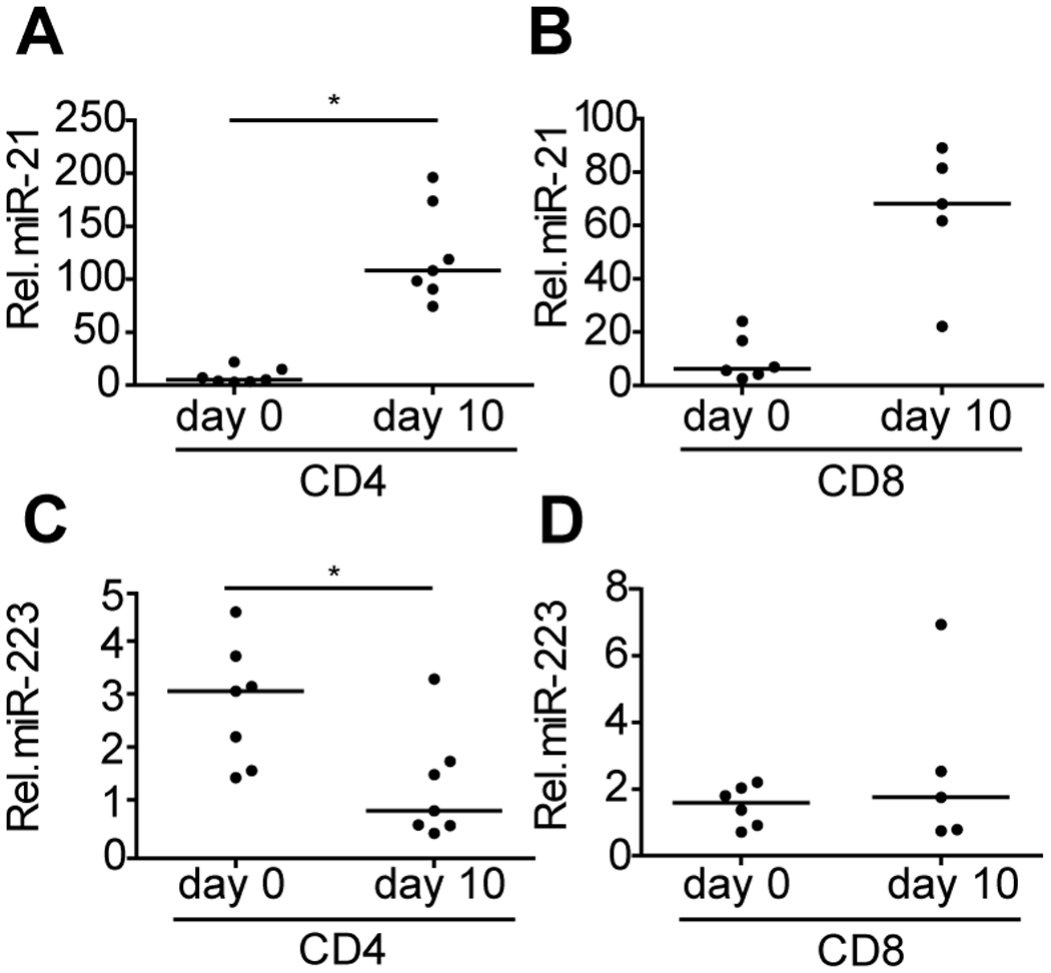
T-cell activation induces upregulation of miR-21 and downregulation of miR-223. Relative expression of miR-21 in stimulated **(A)** CD4+CD45RO- and **(B)** CD4-CD45RO- T cells. Relative expression of miR-223 in stimulated **(C)** CD4+CD45RO- and **(D)** CD4-CD45RO- T cells. MiRNA expression was normalized to the expression of RNU49 (line indicates median). *p<0.05.

Prolonged exposure of T cells to pathogens in elderly increases the number of TCR primed antigen-experienced memory T cells. To explore a relation between the increased miR-21 and miR-223 levels in elderly and T cell activation, we sorted both CD4+ and CD8+ CD45RO- T cells from peripheral blood of 7 healthy young donors (median age 27) (Table A in S1 File) and stimulated these cells with αCD3/αCD28. Stimulation of CD45RO- T cells *in vitro* induced an activated memory phenotype with a marked induction of both CD25 and CD45RO (Figure E in S1 File). A significant induction of miR-21 was observed in CD4+ T cells and a borderline significant induction in CD8+ T cells after stimulation for 10 days (Fig 4A and 4B). Expression of miR-223 was significantly decreased upon αCD3/αCD28 triggering in CD4+ T cells, whereas no difference was observed in CD8+ T cells (Fig 4C and 4D). Thus, our data show that *in vitro* activation of CD4+ T cells is associated with the transgression to the memory phenotype (CD45RO+), induction of miR-21 and reduction of miR-223. *In vitro* activation of CD8+ T cells is also associated with acquisition of the memory phenotype (CD45RO+) and the induction of miR-21, whereas the miR-223 levels remain constant.

### MiR-21 is increased in aged CD4+ T cell clones

**Fig 5:**
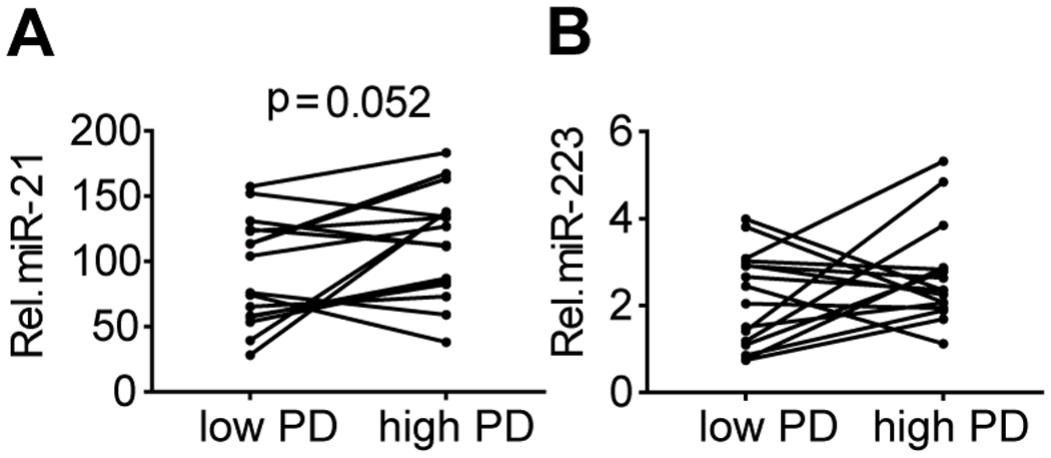
MiR-21 is upregulated with increasing population doublings in T-cell clones. Relative expression of **(A)** miR-21 and **(B)** miR-223 in T cell clones harvested at lower and higher number of population doublings (PD). MiRNA expression was normalized to the expression of RNU49.

We next tested, if besides T-cell composition and activation, we could also identify an age-related component that could contribute to the enhanced miR-21 and miR-223 levels. As an *in vitro* model of T-cell aging, we studied expression of these two miRNAs in 16 CD4+ T-cell clones harvested at a lower (<40) (median population doubling (PD) is 28) and a higher (>40) (median PD is 55) number of PDs. Expression of miR-21 tended to be increased in high vs low PD T-cell clones (p = 0.0525), whereas the miR-223 levels did not differ in T-cell clones with low and high population doublings (Fig 5A and 5B). Thus, based on this *in vitro* model of T-cell ageing, the age-related increase of miR-21 may be associated with increased numbers of cell divisions and continuous activation of the T-cell clones.

## Discussion

In order to decipher age-related differences in miRNA expression patterns we studied CD45RO+ and CD45RO- T cells of healthy young and elderly individuals. We observed clear differences in miRNA expression patterns between CD45RO- and CD45RO+ T cells, consistent with previous studies [21,22]. The next clustering step indicated an age-related difference in CD45RO- T cells and a CD4/CD8 based clustering in CD45RO+ T cells. The pronounced age-related clustering in CD45RO- T cells has not been described previously.

Seventeen miRNAs were found to be ≥2-fold differentially expressed. For miR-15a, miR-21, miR-22 and miR-223 a relation with age was shown previously in studies conducted in humans, mice and rhesus monkeys [23,24]. RNA sequencing of skeletal muscles of old rhesus monkeys revealed age-related upregulation of miR-15a, of which functional involvement in ageing yet remains to be identified [25]. An age-related elevated expression of miR-21 has been described in plasma samples of healthy elderly and geriatric patients with cardiovascular disease and, as such, miR-21 has been established as a circulating marker of inflammageing [26]. Expression of miR-22 was prominently upregulated during cardiac ageing of C57/BI6N mice and overexpression was shown to induce cellular senescence of cardiac fibroblasts [27]. MiR-223 levels were increased in bone marrow derived dendritic cells from aged C57BL/6 mice [28]. Several previously reported age-related miRNA expression changes in T cells were confirmed by our study. Expression levels of the miR-17, miR-19, miR-20a and miR-106a were downregulated in a mix of naïve and memory CD8+CD28+ T cells [13]. We did not find downregulation of these miRNAs in naive CD45RO- T-cell subsets. However, in memory CD8+CD45RO+ T cells we observed a similar age-related downregulation for miR-17, miR-19 and miR-20a, whereas the levels of miR-106a were below detection limit in most samples. In CD4+ T_NAIVE_ cells an age-associated decline of miR-181a expression has been reported [16]. Consistent with this finding we also observed a decreased expression of miR-181a expression with age although this did not reach our 2-fold cut off criterion (1.5 fold). Thus our data substantially increase the list of currently known age-related miRNA expression changes in specific T-cell subsets.

We next assessed if the observed age related differences in miRNA expression patterns are caused by heterogeneity in the composition of the CD45RO- T cells or are truly associated with age. In the CD8 T-cell compartment we showed that the expression levels of miR-21 and miR-223 were strongly upregulated in CD45RO-CCR7- T_EMRA_ vs CD45RO-CCR7+ T_NAIVE_ CD8+ cells. CCR7 is a predicted target for miR-21 and we previously demonstrated that miR-21 regulates expression of CCR7 in CD4+CD45RO- T_NAIVE_ cells following activation [29]. The increased expression of miR-21 in CD8+CD45RO-CCR7- T_EMRA_ cells is thus consistent with its CCR7 targeting potential. There are currently no studies showing a direct link between miR-223 and CCR7. MiRNA target gene prediction algorithms such as TargetScan, PicTar and miRanda also showed no predicted miR-223 binding sites in the CCR7 transcript. In CD4+ T cells, ageing has also been shown to alter T-cell composition, i.e. loss of CD31 a characteristic marker of post-thymic central naïve CD4+ T cells [5]. We observed higher levels of miR-21 in central naive CD4+CD31- T cells compared to CD31+ T_NAIVE_ cells. As miR-21 expression levels are induced upon activation [29] and central naïve CD4+CD31- T cells have a history of antigenic stimulation [30], it might be anticipated that stimulation of CD31+ and CD31- CD4+ T-cell subsets will result in differential induction of miR-21 levels. Target gene prediction programs show no predicted target sites for miR-21 in the 3’-UTR of the CD31 mRNA transcript. Thus, it remains unclear if and how increased miR-21 levels affect CD31 loss. Overall, our data show that ageing-related changes in miRNA expression levels are at least partly induced by changes in T-cell subset composition.

Stimulation of T cells with αCD3/αCD28 results in enhanced miR-21 levels [31]. Consistent with this we showed that miR-21 is highly induced upon TCR triggering, with the most pronounced effects in CD4+ T cells. As the increase of both T_EMRA_ and CD31- T cells in elderly is at least in part related to prolonged stimulation, this might explain the accumulation of miR-21 in these T-cell subsets. The vast majority of CD4 and CD8 CD45RO- T cells in young individuals are CD28+ [32]. Nonetheless, we cannot exclude the possibility that differences in CD28 expression between the CD4 and CD8 populations could influence the effectiveness of the αCD3/CD28 stimulation and thus the degree of miRNA expression increase or decrease.

T_EMRA_ cells clonally expand as a result of chronic antigenic stimulation and their numbers increase upon ageing [33]. CD8+ T_EMRA_ cells re-express CD45RA and lose expression of CD45RO, CD27 and CD28. Hence, T_EMRA_ cells are considered senescent T cells with impaired T-cell functionality [2,34]. Our data show that age-related differences in miR-21 and miR-223 expression levels in CD45RO- T cells may partly reflect the accumulation of CD8+ T_EMRA_ cells in elderly. Post-thymic proliferating central naïve CD31- T cells have undergone TCR stimulation, which might explain the increased miR-21 levels in central naïve T cells. Induction of miR-21 in response to TCR engagement has been reported previously and the levels of miR-21 are increased in inflammatory conditions, such as rheumatoid arthritis [29,35]. Moreover, miR-21 is well characterized in terms of its anti-apoptotic and pro-survival properties in T cells [36,37]. In line with this, miR-21 was shown to act as an oncomiR in a conditional mouse model [38]. Another study showed increased miR-21 levels upon replicative and stress-induced senescence in endothelial cells, suggesting a role for miR-21 in senescence-induced growth arrest [39]. MiR-21 mediates suppression of apoptosis in part by targeting Tipe2 [37]. These data support a role of the TCR-induced increase of miR-21 in expansion and survival of both T_EMRA_ and central naïve T cells in aged individuals. Expression of miR-223 was decreased in naïve CD4+ T cells following αCD3/αCD28 stimulation, whereas levels remained constant in CD8+ T cells. This is in line with the previously reported reduction of miR-223 upon activation of T_NAIVE_ cells [40].

To study whether CD4+ T cell changes in miR-21 and miR-223 could also be related to cellular ageing we used T-cell clones with low and high population doublings as an *in vitro* model of ageing [10]. Extensive inter-clonal heterogeneity made it difficult to establish a clear correlation for miR-223, but there was a borderline significant increase in miR-21 with increasing PD when averaging the results from all the clones. This heterogeneity is not unexpected among monoclonal T-cell populations of different individuals. Next to the proposed role of miR-21 on survival of T_EMRA_ and central naïve T cells, we propose that an age-related increase in miR-21 expression levels may also contribute to the increased miR-21 levels in ageing T cells. MiR-21 was found to induce replicative and stress-induced senescence in human endothelial cells via targeting p21(CIP) and CDC25A cell cycle genes [39]. These T-cell clone experiments thus provide a first indication that changes in levels of miR-21 and potentially also miR-223 measured longitudinally may be related to cellular ageing, but further experiments are required to substantiate this notion.

## Conclusions

Taken together, our results provide an evidence for an age-related miRNA expression pattern especially in CD45RO- T cells. These changes are most likely defined by a combination of ageing-related changes in T-cell composition, activation and the number of cell divisions. MiR-223 changes are associated specifically with an accumulation of T_EMRA_ cells, whereas increase in miR-21 levels are related to the number of cell divisions, the accumulation of T_EMRA_ and CD31- central naïve T cells, the latter likely reflecting post-thymic TCR stimulation.

## Supporting Information

S1 File **Figure A-**Representative scatter plots from T-cell subsets sorting strategies. **Figure B-** T-cell subsets purity following cell sorting. **Figure C-**Heatmap of the differentially expressed miRNAs in naïve (CD45RO-) and memory (CD45RO+) T cells from young and old donors. **Figure D-**Validation of microRNA array. **Figure E-**Activated T cell phenotype. **Table A-**Characteristics of donors. **Table B-**Characteristics of T cell clones. **Table C-**Normalized expression values of miRNAs. **Table D-**Differentially expressed miRNAs in CD45RO- (CD4/CD8) T cells.
(DOC)
