# Supplementary material for: Age-Associated Differences in MiRNA Signatures Are Restricted to CD45RO Negative T Cells and Are Associated with Changes in the Cellular Composition, Activation and Cellular Ageing
Source: PLoS One. 2015 Sep 11;10(9):e0137556. doi: 10.1371/journal.pone.0137556 (PMC4567287; doi:10.1371/journal.pone.0137556)
Supplement: S1 File — Figure A-Representative scatter plots from T-cell subsets sorting strategies. Figure B- T-cell subsets purity following cell sorting. Figure C-Heatmap of the differentially expressed miRNAs in naïve (CD45RO-) and memory (CD45RO+) T cells from young and old donors. Figure D-Validation of microRNA array. Figure E-Activated T cell phenotype. Table A-Characteristics of donors. Table B-Characteristics of T cell clones. Table C-Normalized expression values of miRNAs. Table D-Differentially expressed miRNAs in CD45RO- (CD4/CD8) T cells. (DOC) [file pone.0137556.s001.doc]

**Table A in S1 File. Characteristics of the donors**

| ***Donors*** | ***Gender*** | ***Age (y)*** | ***miRNA analysis*** | ***Data*** |
| --- | --- | --- | --- | --- |
| y1 | M | 26 | array; validation | Fig.1 A-C |
| y2 | F | 28 | array; validation |
| y3 | F | 27 | array; validation |
| y4 | M | 27 | array; validation |
| y5 | M | 28 | array; validation |
| y6 | M | 28 | validation; T cell stimulation | Fig. 1 C  Fig. 4 A-D |
| y7 | F | 28 | validation; T cell stimulation |
| y8 | F | 28 | validation; T cell stimulation |
| y9 | F | 27 | validation; T cell stimulation |
| y10 | M | 24 | validation; T cell stimulation |
| y11 | M | 27 | validation; T cell stimulation |
| y12 | F | 24 | validation; T cell stimulation |
| y13 | F | 27 | T_NAIVE_ / T_EMRA_ | Fig. 2 A-D |
| y14 | F | 26 | T_NAIVE_ / T_EMRA_ |
| y15 | F | 25 | T_NAIVE_ / T_EMRA_ |
| y16 | F | 28 | T_NAIVE_ / T_EMRA_ |
| y17 | M | 27 | T_NAIVE_ / T_EMRA_ |
| y18 | F | 29 | T_NAIVE_ / T_EMRA_ |
| Y19 | M | 27 | T_NAIVE_ / T_EMRA_ |
| y20 | F | 28 | CD31+ / CD31- | Fig. 3 A-D |
| y21 | F | 22 | CD31+ / CD31- |
| y22 | F | 29 | CD31+ / CD31- |
| y23 | M | 23 | CD31+ / CD31- |
| y24 | F | 28 | CD31+ / CD31- |
| y25 | F | 27 | CD31+ / CD31- |
| y26 | M | 28 | CD31+ / CD31- |
| y27 | F | 21 | CD31+ / CD31- |
| o1 | M | 62 | array; validation | Fig. 1 A-C  Fig. 2 A-D |
| o2 | F | 71 | array; validation |
| o3 | F | 56 | array; validation; T_NAIVE_ / T_EMRA_ |
| o4 | F | 67 | array; validation ;T_NAIVE_ / T_EMRA_ |
| o5 | F | 64 | array; validation; T_NAIVE_ / T_EMRA_ |
| o6 | F | 62 | validation | Fig. 1 C |
| o7 | M | 65 | validation |
| o8 | F | 67 | validation |
| o9 | F | 72 | validation |
| o10 | F | 61 | validation |
| o11 | M | 68 | validation |
| o12 | F | 65 | validation |
| o13 | F | 72 | validation |
| o14 | F | 71 | T_NAIVE_ / T_EMRA_ | Fig. 2. A-D |
| o15 | F | 76 | T_NAIVE_ / T_EMRA_ |
| o16 | F | 81 | T_NAIVE_ / T_EMRA_ |
| o17 | M | 64 | T_NAIVE_ / T_EMRA_ |
| o18 | F | 77 | CD31+ / CD31- | Fig. 3 A-D |
| o19 | M | 72 | CD31+ / CD31- |
| o20 | F | 82 | CD31+ / CD31- |
| o21 | M | 66 | CD31+ / CD31- |
| o22 | F | 75 | CD31+ / CD31- |
| o23 | M | 75 | CD31+ / CD31- |
| o24 | F | 70 | CD31+ / CD31- |

Y= young donors, o= old donors

**Table B in S1 File. Characteristics of the T cell clones**

| ***Donors*** | ***Age (y)*** | ***Clone*** | ***Low PD*** | ***High PD*** | |
| --- | --- | --- | --- | --- | --- |
| 1 | 31 | 454-6  456-25  256-4  256-4 | 28  27  26  25 | 66  65  56  54 |  |
| 2 | >85 (L12) | 433-26  433-6  433-9  433-25 | 26  28  32  36 | 61  66  48  55 |  |
| 3 | >85 (L15) | 434-29 | 28 | 41 |  |
| 4 | 100 (887) | 461-15  461-30  461-17  461-23  461-33 | 27  27  39  35  32 | 64  68  68  54  73 |  |
| 5 | 100 (842) | 460-31  460-38 | 30  29 | 48  45 |  |

**Table C in S1 File.** Normalized expression values of miRNAs

| ***# miRNA*** | ***Young naive CD4*** | ***Young naive CD8*** | ***Old naive CD4*** | ***Old naive CD8*** | ***Young memory CD4*** | ***Young memory CD8*** | ***Old memory CD4*** | ***Old memory CD8*** |
| --- | --- | --- | --- | --- | --- | --- | --- | --- |
| hsa-let-7a | 40,1 | 32,9 | 49,7 | 47,0 | 32,5 | 38,2 | 41,1 | 45,5 |
| hsa-let-7b | 18,7 | 12,6 | 23,9 | 16,3 | 20,2 | 13,8 | 30,6 | 21,0 |
| hsa-let-7b* | 0,8 | 1,0 | 0,6 | 0,5 | 0,8 | 0,6 | 0,6 | 0,9 |
| hsa-let-7c | 2,2 | 1,3 | 3,1 | 2,2 | 2,3 | 1,5 | 3,3 | 2,4 |
| hsa-let-7d | 6,8 | 6,1 | 7,3 | 6,9 | 6,7 | 6,3 | 6,8 | 6,6 |
| hsa-let-7d* | 0,5 | 0,6 | 0,2 | 0,3 | 0,4 | 0,1 | 0,3 | 0,4 |
| hsa-let-7e | 0,5 | 0,2 | 0,5 | 0,4 | 0,7 | 0,4 | 0,9 | 0,6 |
| hsa-let-7f | 24,4 | 22,3 | 35,1 | 39,1 | 22,6 | 32,5 | 29,9 | 37,1 |
| hsa-let-7f-1* | 0,6 | 0,7 | 0,5 | 0,5 | 0,7 | 0,6 | 0,6 | 0,6 |
| hsa-let-7g | 76,6 | 76,2 | 104,5 | 92,4 | 64,1 | 76,6 | 77,8 | 79,1 |
| hsa-let-7i | 11,7 | 13,6 | 16,5 | 20,1 | 20,0 | 23,3 | 23,9 | 22,8 |
| hsa-miR-101 | 1,1 | 1,3 | 1,6 | 1,8 | 0,8 | 1,4 | 1,1 | 1,2 |
| hsa-miR-103 | 5,6 | 7,7 | 7,3 | 10,1 | 7,6 | 12,5 | 7,9 | 10,9 |
| hsa-miR-106b | 2,2 | 3,0 | 2,3 | 3,9 | 2,5 | 4,3 | 2,8 | 3,6 |
| hsa-miR-107 | 4,3 | 5,1 | 5,5 | 7,4 | 6,8 | 8,2 | 6,6 | 8,7 |
| hsa-miR-1224-3p | 0,8 | 0,4 | 0,2 | 0,3 | 0,8 | 0,0 | 0,6 | 0,5 |
| hsa-miR-1225-3p | 0,7 | 0,8 | 0,7 | 0,5 | 0,9 | 0,7 | 0,8 | 0,7 |
| hsa-miR-1225-5p | 1,2 | 1,7 | 1,4 | 1,2 | 1,5 | 1,6 | 1,6 | 1,5 |
| hsa-miR-1227 | 0,4 | 0,5 | 0,3 | 0,3 | 0,4 | 0,2 | 0,3 | 0,4 |
| hsa-miR-1228 | 0,9 | 1,0 | 0,9 | 0,7 | 1,1 | 1,2 | 1,1 | 1,0 |
| hsa-miR-1229 | 0,4 | 0,4 | 0,3 | 0,3 | 0,5 | 0,3 | 0,4 | 0,3 |
| hsa-miR-1234 | 1,3 | 1,3 | 1,1 | 0,8 | 1,4 | 1,1 | 1,2 | 1,1 |
| hsa-miR-1236 | 0,3 | 0,4 | 0,1 | 0,2 | 0,3 | 0,1 | 0,2 | 0,3 |
| hsa-miR-1237 | 0,5 | 0,6 | 0,5 | 0,4 | 0,5 | 0,5 | 0,6 | 0,6 |
| hsa-miR-1238 | 0,8 | 1,0 | 0,8 | 0,6 | 1,1 | 0,9 | 1,0 | 0,9 |
| hsa-miR-125a-5p | 0,5 | 0,2 | 0,5 | 0,3 | 0,8 | 0,3 | 0,9 | 0,3 |
| hsa-miR-125b | 1,9 | 0,3 | 1,7 | 1,2 | 0,2 | 0,0 | 0,2 | 0,0 |
| hsa-miR-126 | 0,1 | 0,2 | 0,5 | 0,6 | 0,1 | 0,3 | 0,4 | 0,4 |
| hsa-miR-128 | 0,2 | 0,1 | 0,2 | 0,3 | 0,1 | 0,2 | 0,2 | 0,4 |
| hsa-miR-130b | 0,1 | 0,1 | 0,2 | 0,3 | 0,4 | 0,2 | 0,4 | 0,4 |
| hsa-miR-139-3p | 0,2 | 0,2 | 0,3 | 0,1 | 0,2 | 0,1 | 0,2 | 0,1 |
| hsa-miR-140-3p | 3,9 | 3,7 | 3,7 | 4,5 | 4,5 | 4,9 | 3,9 | 4,4 |
| hsa-miR-140-5p | 1,5 | 1,8 | 1,5 | 2,5 | 1,5 | 2,7 | 1,5 | 2,9 |
| hsa-miR-142-3p | 27,0 | 36,3 | 47,9 | 63,5 | 27,2 | 47,6 | 36,8 | 68,8 |
| hsa-miR-142-5p | 2,9 | 4,1 | 3,6 | 5,4 | 2,8 | 5,5 | 3,1 | 4,9 |
| hsa-miR-146a | 2,2 | 1,5 | 2,0 | 4,9 | 10,7 | 6,1 | 10,6 | 9,8 |
| hsa-miR-146b-5p | 9,2 | 14,3 | 12,3 | 12,1 | 6,3 | 5,1 | 7,3 | 4,8 |
| hsa-miR-148a | 0,2 | 0,1 | 0,2 | 0,3 | 0,2 | 0,4 | 0,3 | 0,3 |
| hsa-miR-149 | 0,8 | 0,9 | 0,5 | 0,5 | 0,9 | 0,6 | 0,6 | 0,8 |
| hsa-miR-150 | 583,1 | 366,2 | 549,4 | 327,7 | 584,3 | 334,0 | 603,1 | 316,3 |
| hsa-miR-150* | 0,1 | 0,1 | 0,1 | 0,1 | 0,1 | 0,1 | 0,2 | 0,1 |
| hsa-miR-151-5p | 1,8 | 0,4 | 1,3 | 0,6 | 0,5 | 0,2 | 0,5 | 0,1 |
| hsa-miR-155 | 2,1 | 1,6 | 2,4 | 2,5 | 5,9 | 5,3 | 7,0 | 6,5 |
| hsa-miR-15a | 2,3 | 2,3 | 4,1 | 6,6 | 4,9 | 9,1 | 6,8 | 12,7 |
| hsa-miR-15b | 17,0 | 12,2 | 12,4 | 15,2 | 24,5 | 19,3 | 21,9 | 20,3 |
| hsa-miR-16 | 26,9 | 21,7 | 29,7 | 39,5 | 49,3 | 48,0 | 53,2 | 51,0 |
| hsa-miR-17 | 3,8 | 4,4 | 4,0 | 4,0 | 3,8 | 4,2 | 3,6 | 3,0 |
| hsa-miR-181a | 7,1 | 6,7 | 5,0 | 4,1 | 3,7 | 2,9 | 2,7 | 3,2 |
| hsa-miR-181b | 1,2 | 1,3 | 0,8 | 1,0 | 0,9 | 0,8 | 0,8 | 0,8 |
| hsa-miR-181d | 0,7 | 0,7 | 0,3 | 0,3 | 0,6 | 0,6 | 0,3 | 0,2 |
| hsa-miR-185 | 0,2 | 0,3 | 0,4 | 0,7 | 0,4 | 0,8 | 0,4 | 0,7 |
| hsa-miR-186 | 0,6 | 0,6 | 0,6 | 0,9 | 0,5 | 0,8 | 0,6 | 0,9 |
| hsa-miR-191* | 0,7 | 1,0 | 0,7 | 0,5 | 0,8 | 0,8 | 0,9 | 0,8 |
| hsa-miR-192 | 0,9 | 1,1 | 0,8 | 1,0 | 0,8 | 1,0 | 0,8 | 0,9 |
| hsa-miR-193b | 0,6 | 0,4 | 0,5 | 0,3 | 0,1 | 0,0 | 0,0 | 0,2 |
| hsa-miR-194 | 0,2 | 0,2 | 0,3 | 0,3 | 0,2 | 0,3 | 0,2 | 0,3 |
| hsa-miR-195* | 0,3 | 0,3 | 0,1 | 0,2 | 0,3 | 0,1 | 0,2 | 0,3 |
| hsa-miR-196a | 0,1 | 0,1 | 0,1 | 0,3 | 0,2 | 0,2 | 0,1 | 0,2 |
| hsa-miR-197 | 9,0 | 4,9 | 3,3 | 2,6 | 5,9 | 3,8 | 4,5 | 3,9 |
| hsa-miR-199b-3p | 0,1 | 0,2 | 0,6 | 0,5 | 0,1 | 0,2 | 0,3 | 0,3 |
| hsa-miR-19a | 1,6 | 2,3 | 1,9 | 2,7 | 1,4 | 2,7 | 1,6 | 1,9 |
| hsa-miR-19b | 12,7 | 14,8 | 11,7 | 14,2 | 9,3 | 14,3 | 10,0 | 10,0 |
| hsa-miR-20a | 10,3 | 14,6 | 11,6 | 12,3 | 8,6 | 11,5 | 9,6 | 8,5 |
| hsa-miR-20b | 1,9 | 1,8 | 2,1 | 1,8 | 1,0 | 1,2 | 1,2 | 1,1 |
| hsa-miR-21 | 13,6 | 17,1 | 25,8 | 36,6 | 65,6 | 40,6 | 82,1 | 64,9 |
| hsa-miR-21* | 0,1 | 0,1 | 0,2 | 0,2 | 0,7 | 0,4 | 0,5 | 0,3 |
| hsa-miR-211 | 0,5 | 0,6 | 0,2 | 0,3 | 0,4 | 0,1 | 0,2 | 0,5 |
| hsa-miR-212 | 0,6 | 0,7 | 0,6 | 0,6 | 0,6 | 0,7 | 0,4 | 0,7 |
| hsa-miR-22 | 0,7 | 2,2 | 1,9 | 5,4 | 4,5 | 8,9 | 4,5 | 9,5 |
| hsa-miR-220b | 0,0 | 0,1 | 0,0 | 0,1 | 0,1 | 0,0 | 0,1 | 0,0 |
| hsa-miR-221 | 0,2 | 0,1 | 0,3 | 0,3 | 0,2 | 0,3 | 0,4 | 0,4 |
| hsa-miR-222 | 0,2 | 0,2 | 0,1 | 0,4 | 0,9 | 0,5 | 0,7 | 0,6 |
| hsa-miR-223 | 2,7 | 2,5 | 6,0 | 4,8 | 3,4 | 4,1 | 4,1 | 4,8 |
| hsa-miR-23a | 3,6 | 4,2 | 5,8 | 8,7 | 8,5 | 14,5 | 9,0 | 15,7 |
| hsa-miR-23b | 0,5 | 0,4 | 0,5 | 0,7 | 0,4 | 0,7 | 0,6 | 0,8 |
| hsa-miR-24 | 2,8 | 4,3 | 5,1 | 8,0 | 10,3 | 15,6 | 9,9 | 15,3 |
| hsa-miR-25 | 5,5 | 6,1 | 6,5 | 7,5 | 5,7 | 6,9 | 6,0 | 7,0 |
| hsa-miR-26a | 19,2 | 15,7 | 21,3 | 18,2 | 14,2 | 14,4 | 14,7 | 13,4 |
| hsa-miR-26b | 11,2 | 12,3 | 13,5 | 23,3 | 12,9 | 23,9 | 13,3 | 23,8 |
| hsa-miR-27a | 0,8 | 1,6 | 1,6 | 2,5 | 2,5 | 4,8 | 2,7 | 4,9 |
| hsa-miR-27b | 0,2 | 0,2 | 0,3 | 0,5 | 0,3 | 0,5 | 0,3 | 0,5 |
| hsa-miR-28-5p | 0,7 | 1,0 | 0,9 | 2,2 | 1,2 | 2,4 | 1,4 | 2,2 |
| hsa-miR-296-5p | 0,4 | 0,4 | 0,3 | 0,2 | 0,4 | 0,4 | 0,3 | 0,4 |
| hsa-miR-29a | 26,4 | 30,7 | 30,5 | 40,9 | 41,0 | 44,2 | 38,1 | 38,6 |
| hsa-miR-29b | 2,8 | 4,6 | 5,3 | 7,5 | 4,9 | 8,6 | 6,0 | 7,8 |
| hsa-miR-29b-1* | 0,1 | 0,2 | 0,3 | 0,3 | 0,4 | 0,4 | 0,5 | 0,4 |
| hsa-miR-29c | 9,1 | 11,6 | 12,3 | 13,2 | 6,8 | 9,7 | 8,2 | 9,2 |
| hsa-miR-29c* | 0,3 | 0,3 | 0,1 | 0,3 | 0,2 | 0,1 | 0,3 | 0,3 |
| hsa-miR-30b | 5,2 | 6,3 | 5,6 | 6,0 | 4,0 | 5,2 | 3,8 | 4,7 |
| hsa-miR-30c | 1,9 | 1,7 | 1,4 | 1,8 | 1,5 | 1,9 | 1,6 | 1,9 |
| hsa-miR-30d | 1,4 | 1,4 | 1,3 | 1,4 | 1,1 | 1,5 | 1,2 | 1,4 |
| hsa-miR-30e | 1,3 | 1,9 | 1,6 | 2,5 | 1,2 | 2,5 | 1,4 | 2,3 |
| hsa-miR-30e* | 0,3 | 0,3 | 0,4 | 0,5 | 0,3 | 0,4 | 0,2 | 0,5 |
| hsa-miR-31 | 0,8 | 1,6 | 1,2 | 1,0 | 0,4 | 0,4 | 0,8 | 0,4 |
| hsa-miR-32* | 0,8 | 0,2 | 0,2 | 0,1 | 0,8 | 0,0 | 0,9 | 0,5 |
| hsa-miR-320 | 2,6 | 3,4 | 3,0 | 2,3 | 2,2 | 2,3 | 2,3 | 2,0 |
| hsa-miR-324-3p | 2,9 | 3,1 | 3,0 | 2,3 | 3,5 | 3,4 | 3,2 | 3,1 |
| hsa-miR-328 | 1,5 | 1,0 | 0,4 | 0,6 | 1,3 | 0,2 | 0,8 | 1,1 |
| hsa-miR-331-3p | 1,8 | 2,0 | 1,4 | 1,9 | 2,8 | 2,4 | 2,1 | 2,4 |
| hsa-miR-33b* | 0,4 | 0,6 | 0,5 | 0,3 | 0,6 | 0,5 | 0,6 | 0,6 |
| hsa-miR-342-3p | 80,0 | 58,5 | 68,7 | 50,8 | 56,1 | 48,7 | 58,2 | 45,1 |
| hsa-miR-342-5p | 8,2 | 7,0 | 8,5 | 5,2 | 6,4 | 4,9 | 6,4 | 4,8 |
| hsa-miR-34a | 0,1 | 0,2 | 0,2 | 0,3 | 0,3 | 0,3 | 1,1 | 0,6 |
| hsa-miR-34b | 0,8 | 0,3 | 0,1 | 0,1 | 0,7 | 0,0 | 0,3 | 0,4 |
| hsa-miR-361-3p | 1,4 | 1,4 | 1,6 | 1,9 | 1,7 | 1,7 | 1,7 | 1,6 |
| hsa-miR-361-5p | 1,0 | 0,9 | 1,2 | 1,2 | 0,8 | 1,1 | 0,9 | 1,2 |
| hsa-miR-363 | 2,2 | 1,4 | 1,8 | 1,1 | 0,3 | 0,6 | 0,5 | 0,4 |
| hsa-miR-365 | 0,5 | 0,5 | 0,5 | 0,3 | 0,4 | 0,2 | 0,3 | 0,2 |
| hsa-miR-374a | 0,3 | 0,6 | 0,5 | 1,1 | 0,5 | 1,2 | 0,6 | 1,0 |
| hsa-miR-374b | 0,4 | 0,4 | 0,5 | 0,9 | 0,9 | 1,1 | 0,8 | 1,0 |
| hsa-miR-423-5p | 1,1 | 1,0 | 1,5 | 1,0 | 1,2 | 1,1 | 1,5 | 1,1 |
| hsa-miR-425 | 0,9 | 1,0 | 1,0 | 1,5 | 1,4 | 2,0 | 1,3 | 1,7 |
| hsa-miR-425* | 0,6 | 0,9 | 0,6 | 0,5 | 0,8 | 0,7 | 0,7 | 0,7 |
| hsa-miR-451 | 2,2 | 2,6 | 8,0 | 9,6 | 2,3 | 3,3 | 7,8 | 8,4 |
| hsa-miR-454 | 0,0 | 0,1 | 0,1 | 0,3 | 0,3 | 0,2 | 0,3 | 0,3 |
| hsa-miR-483-3p | 1,4 | 1,1 | 0,4 | 0,5 | 1,4 | 0,2 | 1,0 | 1,1 |
| hsa-miR-484 | 0,3 | 0,3 | 0,2 | 0,3 | 0,4 | 0,3 | 0,4 | 0,4 |
| hsa-miR-485-3p | 0,7 | 0,6 | 0,2 | 0,3 | 0,7 | 0,2 | 0,6 | 0,6 |
| hsa-miR-486-5p | 0,1 | 0,2 | 0,3 | 0,2 | 0,2 | 0,1 | 0,4 | 0,2 |
| hsa-miR-494 | 3,5 | 3,2 | 4,5 | 2,9 | 3,8 | 4,4 | 14,4 | 4,6 |
| hsa-miR-549 | 0,0 | 0,1 | 0,0 | 0,0 | 0,1 | 0,0 | 0,0 | 0,6 |
| hsa-miR-550 | 0,4 | 0,6 | 0,5 | 0,4 | 0,5 | 0,5 | 0,6 | 0,5 |
| hsa-miR-563 | 0,3 | 0,5 | 0,3 | 0,2 | 0,3 | 0,5 | 0,3 | 0,4 |
| hsa-miR-564 | 0,6 | 0,8 | 1,0 | 0,5 | 0,8 | 0,7 | 0,8 | 0,5 |
| hsa-miR-572 | 0,0 | 0,1 | 0,1 | 0,2 | 0,1 | 0,1 | 0,1 | 0,1 |
| hsa-miR-574-3p | 15,1 | 9,6 | 5,1 | 3,8 | 11,3 | 7,7 | 9,4 | 5,8 |
| hsa-miR-574-5p | 4,2 | 0,9 | 1,3 | 0,7 | 3,9 | 0,6 | 3,5 | 2,0 |
| hsa-miR-575 | 0,8 | 0,7 | 0,8 | 0,6 | 0,9 | 0,6 | 0,9 | 0,7 |
| hsa-miR-586 | 0,0 | 0,1 | 0,0 | 0,0 | 0,1 | 0,0 | 0,0 | 0,2 |
| hsa-miR-590-5p | 0,2 | 0,4 | 0,2 | 0,6 | 0,2 | 0,6 | 0,2 | 0,4 |
| hsa-miR-595 | 1,0 | 0,7 | 0,3 | 0,3 | 1,0 | 0,3 | 0,6 | 0,5 |
| hsa-miR-602 | 0,3 | 0,6 | 0,3 | 0,3 | 0,5 | 0,6 | 0,4 | 0,5 |
| hsa-miR-613 | 0,4 | 0,4 | 0,1 | 0,2 | 0,3 | 0,1 | 0,1 | 0,3 |
| hsa-miR-615-3p | 0,3 | 0,4 | 0,1 | 0,2 | 0,3 | 0,1 | 0,3 | 0,3 |
| hsa-miR-623 | 0,4 | 0,3 | 0,1 | 0,1 | 0,3 | 0,1 | 0,1 | 0,2 |
| hsa-miR-625 | 0,9 | 0,8 | 0,9 | 0,8 | 0,9 | 0,8 | 1,0 | 1,0 |
| hsa-miR-631 | 0,4 | 0,3 | 0,1 | 0,1 | 0,3 | 0,0 | 0,2 | 0,2 |
| hsa-miR-634 | 0,3 | 0,3 | 0,1 | 0,1 | 0,2 | 0,2 | 0,3 | 0,3 |
| hsa-miR-638 | 1,5 | 1,8 | 1,7 | 1,4 | 1,9 | 1,5 | 1,7 | 1,5 |
| hsa-miR-647 | 0,5 | 0,2 | 0,0 | 0,1 | 0,4 | 0,0 | 0,2 | 0,2 |
| hsa-miR-652 | 0,1 | 0,1 | 0,2 | 0,2 | 0,1 | 0,3 | 0,1 | 0,2 |
| hsa-miR-654-3p | 0,6 | 0,3 | 0,1 | 0,1 | 0,4 | 0,0 | 0,5 | 0,3 |
| hsa-miR-660 | 0,1 | 0,1 | 0,2 | 0,3 | 0,1 | 0,1 | 0,1 | 0,1 |
| hsa-miR-7 | 0,2 | 0,4 | 0,3 | 0,7 | 0,3 | 0,6 | 0,3 | 0,6 |
| hsa-miR-7-1* | 0,3 | 0,4 | 0,4 | 0,5 | 0,3 | 0,5 | 0,3 | 0,4 |
| hsa-miR-744* | 0,6 | 0,4 | 0,1 | 0,1 | 0,2 | 0,3 | 0,1 | 0,1 |
| hsa-miR-766 | 3,6 | 2,5 | 1,6 | 1,4 | 2,6 | 2,1 | 2,3 | 1,8 |
| hsa-miR-768-3p | 153,1 | 161,5 | 186,6 | 134,7 | 193,8 | 193,8 | 182,6 | 160,0 |
| hsa-miR-768-5p | 4,5 | 3,6 | 4,0 | 3,3 | 4,7 | 4,1 | 4,1 | 3,8 |
| hsa-miR-769-5p | 0,3 | 0,5 | 0,2 | 0,3 | 0,2 | 0,4 | 0,3 | 0,3 |
| hsa-miR-801 | 0,5 | 0,4 | 0,5 | 0,3 | 0,5 | 0,5 | 0,7 | 0,6 |
| hsa-miR-874 | 0,4 | 0,5 | 0,5 | 0,3 | 0,3 | 0,4 | 0,4 | 0,2 |
| hsa-miR-877* | 0,8 | 0,6 | 0,4 | 0,3 | 0,9 | 0,4 | 0,7 | 0,6 |
| hsa-miR-885-5p | 1,3 | 0,8 | 0,3 | 0,4 | 1,0 | 0,1 | 0,6 | 0,7 |
| hsa-miR-886-3p | 0,0 | 0,1 | 0,1 | 0,2 | 0,2 | 0,3 | 0,1 | 0,3 |
| hsa-miR-892b | 0,3 | 0,4 | 0,6 | 0,3 | 0,6 | 0,4 | 0,4 | 0,4 |
| hsa-miR-923 | 92,8 | 138,1 | 213,5 | 134,2 | 145,1 | 170,7 | 338,5 | 240,5 |
| hsa-miR-92a | 13,2 | 10,5 | 14,1 | 8,4 | 8,9 | 7,2 | 9,8 | 6,7 |
| hsa-miR-92b | 0,3 | 0,7 | 0,3 | 0,3 | 0,4 | 0,3 | 0,3 | 0,5 |
| hsa-miR-93 | 0,9 | 0,9 | 1,2 | 1,7 | 1,4 | 1,9 | 1,6 | 1,8 |
| hsa-miR-933 | 0,4 | 0,5 | 0,3 | 0,2 | 0,4 | 0,5 | 0,3 | 0,4 |
| hsa-miR-937 | 0,5 | 0,4 | 0,2 | 0,2 | 0,5 | 0,1 | 0,3 | 0,4 |
| hsa-miR-939 | 1,0 | 1,5 | 1,3 | 1,3 | 1,9 | 2,2 | 1,4 | 1,8 |
| hsa-miR-940 | 1,2 | 1,6 | 1,2 | 1,0 | 1,5 | 1,6 | 1,4 | 1,5 |
| hsa-miR-98 | 0,2 | 0,2 | 0,3 | 0,4 | 0,2 | 0,4 | 0,3 | 0,4 |
| hsa-miR-99a | 0,8 | 0,1 | 0,5 | 0,5 | 0,1 | 0,0 | 0,0 | 0,0 |

**Table D in S1 File.** Normalized expression values of the differentially expressed miRNAs

|  |  |  | |  |
| --- | --- | --- | --- | --- |
| ***# miRNA*** | ***Young***  ***vs old*** | ***Norm. expr. young*** | ***Norm. expr. old*** | ***Fold change*** |
| **hsa-miR-574-3p** | **Up** | **12,4** | **4,5** | **2,7** |
| **hsa-miR-197** | **Up** | **6,8** | **3** | **2,3** |
| **hsa-miR-766** | **Up** | **3** | **1,5** | **2** |
| **hsa-miR-574-5p** | **Up** | **2** | **1,9** | **2** |
| **hsa-miR-328** | **Up** | **1,3** | **0,4** | **3,4** |
| **hsa-miR-483-3p** | **Up** | **1,2** | **0,43** | **3,5** |
| **hsa-miR-885-5p** | **Up** | **1** | **0,4** | **2,3** |
| hsa-miR-181d | Up | 0,7 | 0,2 | 3,7 |
| hsa-miR-595 | Up | 0,7 | 0,1 | 4,3 |
| miR-877* | Up | 0,5 | 0,1 | 3,2 |
| hsa-miR-1224-3p | Up | 0,3 | 0,1 | 3,5 |
| **hsa-miR-21** | **Down** | **15,6** | **31,5** | **2** |
| **hsa-miR-223** | **Down** | **2,6** | **5,5** | **2** |
| **hsa-miR-451** | **Down** | **2,5** | **9** | **3,6** |
| **hsa-miR-15a** | **Down** | **2,3** | **5,3** | **2,3** |
| **hsa-miR-22** | **Down** | **1,1** | **3,3** | **2,8** |
| **hsa-miR-28-5p** | **Down** | **0,7** | **1,4** | **2,1** |

Selected miRNAs are highlighted in bold

**
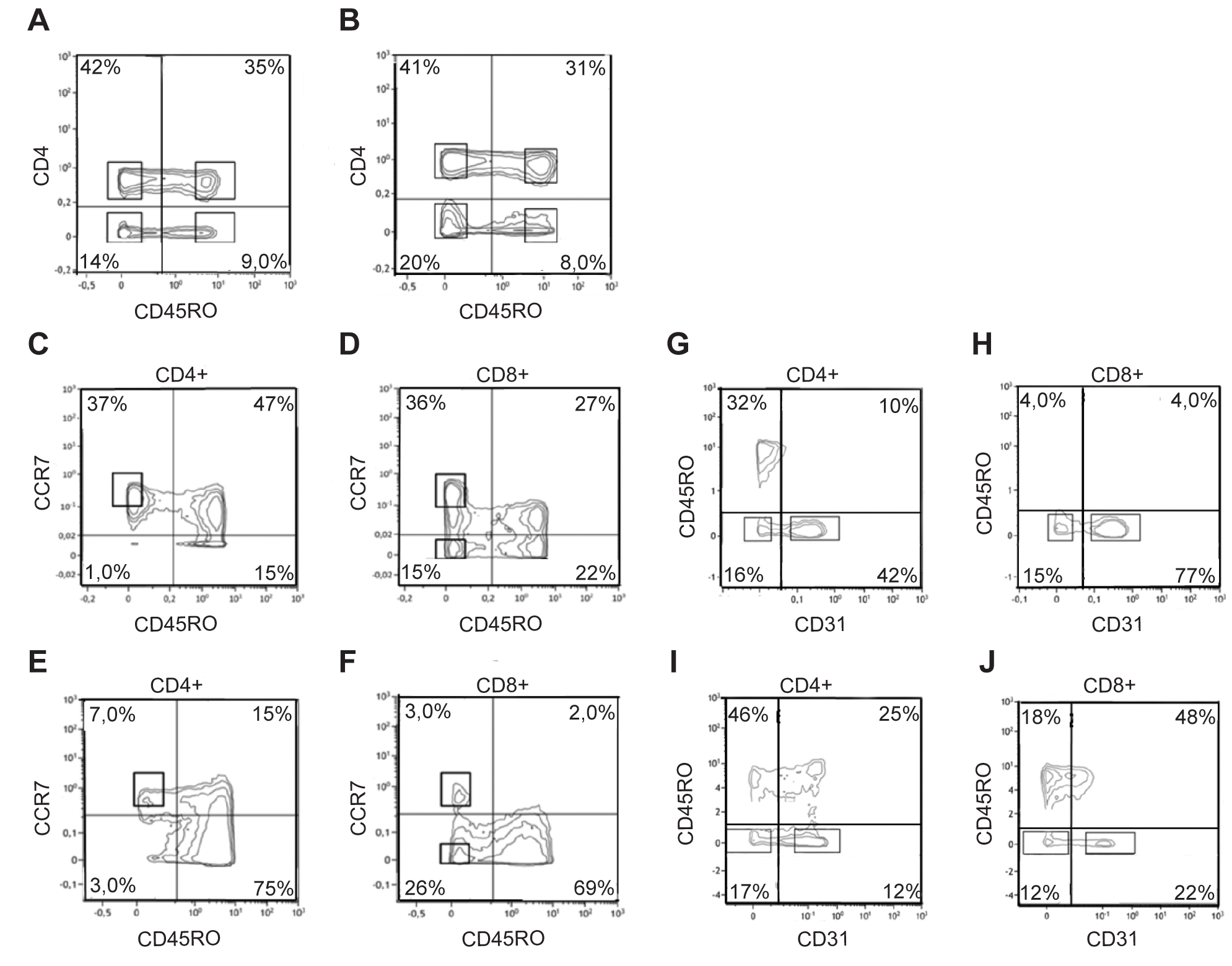
**

**Figure A in S1 File. Representative scatter plots demonstrating T cell subsets sorting strategies.** Sorting of CD45RO- T cells from **(A)** young and (**B)** old donors. Sorting was performed on blood samples from 25 donors; young (n=12) / old (n=13). T_NAIVE_ (CD4/CD8) and T_EMRA_ (CD8) sorting based on CCR7 and CD45RO expression from **(C), (D)** young and **(E), (F)** old donors; representative examples are shown. Sorting was performed on blood samples from 15 donors; young (n=7) / old (n=7). Sorting of CD31+ and CD31- (CD4/CD8) (CD45RO-) T cells from **(G), (H)** young and **(I), (J)** old donors. Sorting was performed on blood samples from 15 donors; young (n=8) / old (n=7).


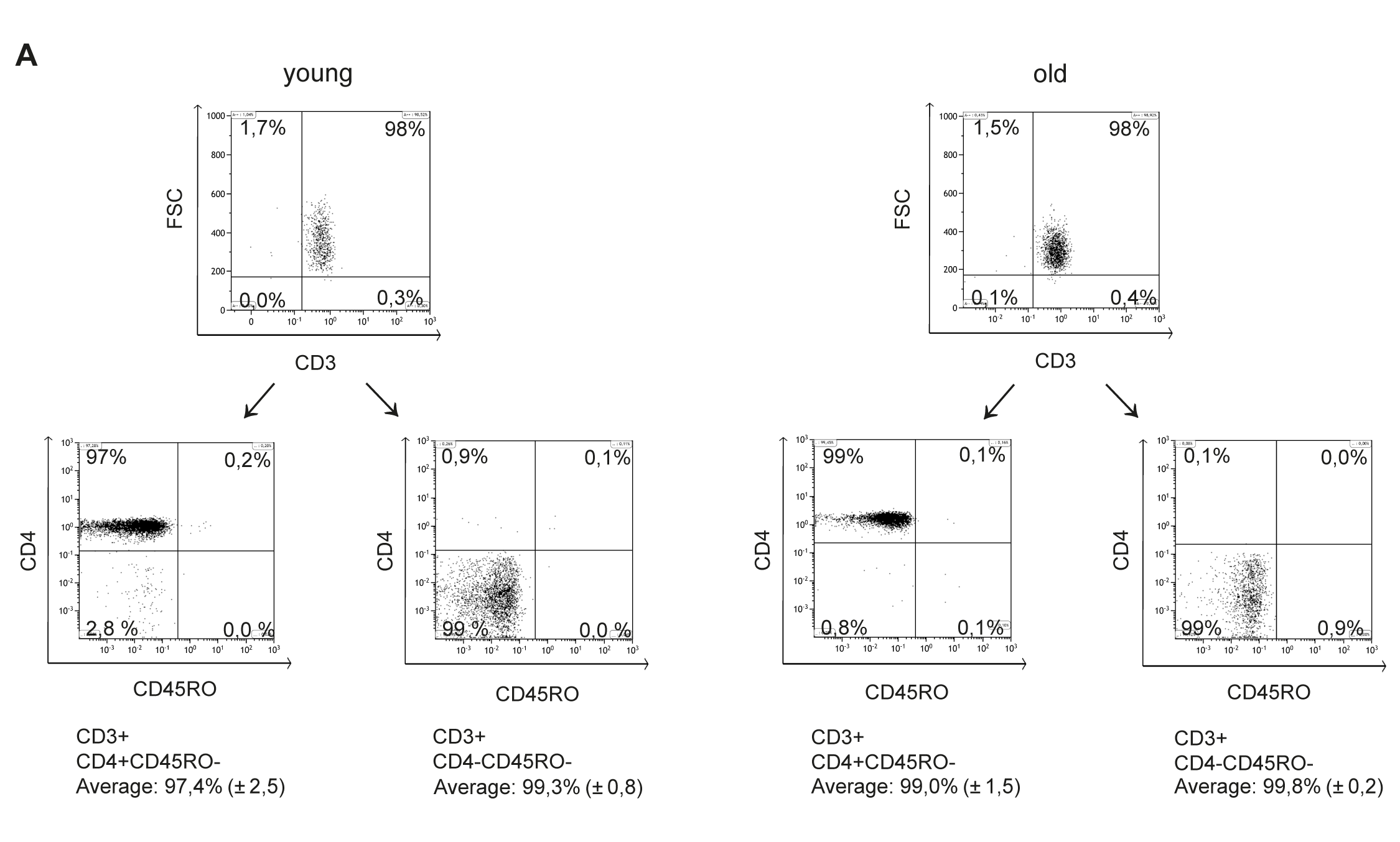


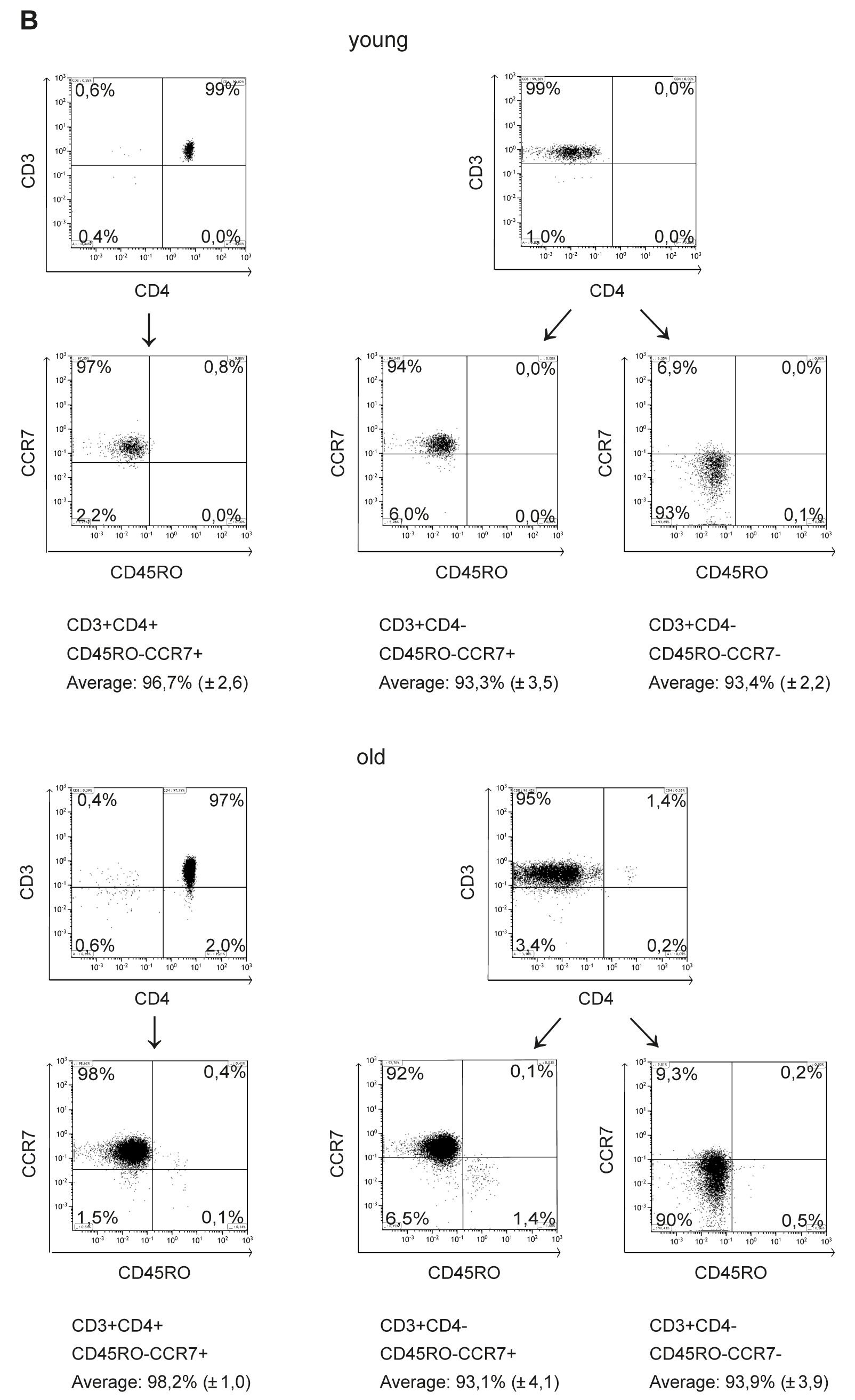


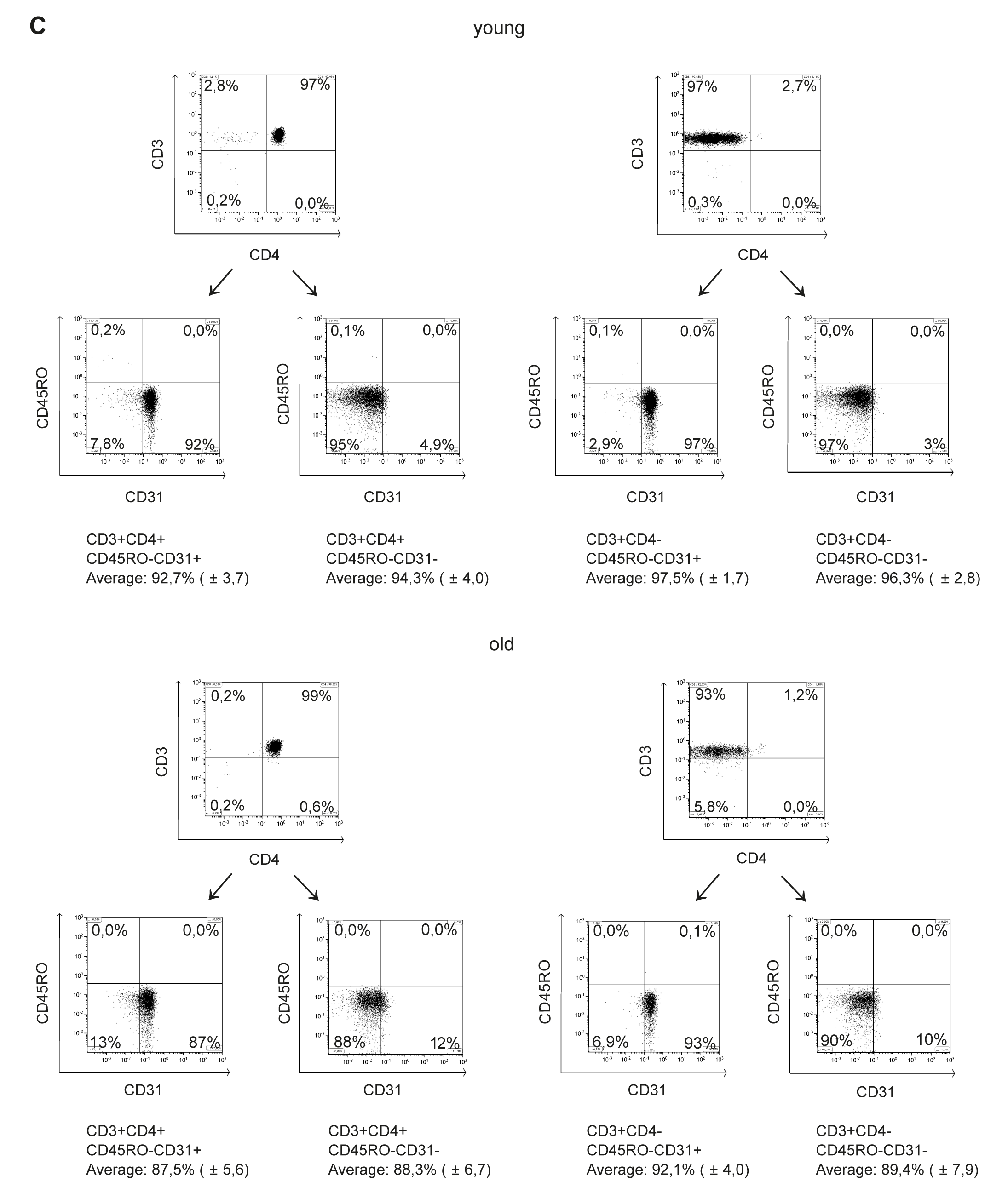


**Figure B in S1 File. Representative scatter plots demonstrating T cell subsets purity following cell sorting.**  **(A)** CD3+CD4+CD45RO- and CD3+CD4-CD45RO- sorted cells from young and old **(B)** CD3+CD4+CD45RO-CCR7+, CD3+CD4-CD45RO-CCR7+ and CD3+CD4-CD45RO-CCR7- sorted cells from young and old **(C)** CD3+CD4+CD45RO-CD31+, CD3+CD4+CD45RO-CD31-, CD3+CD4-CD45RO-CD31+ and CD3+CD4-CD45RO-CD31- sorted cells from young and old. The average percentages (%) with standard deviation (SD) of the sorted T cell population purities are indicated below the scatter plots.


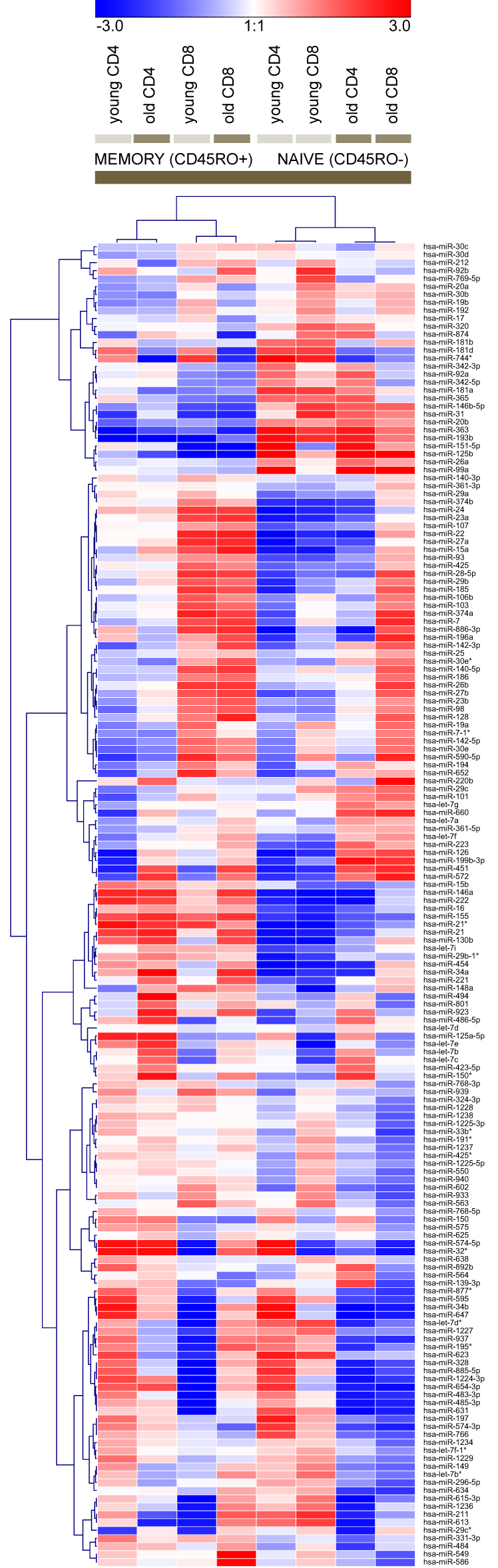


**Figure C in S1 File. MiRNA expression signature characterizing naïve (CD45RO-) and memory (CD45RO+) T cells from young and old CD4 and CD8 T cell subsets.** Unsupervised hierarchical clustering of the 166 miRNAs detected in at least 7 out of 8 samples shows a first separation between CD45RO- and CD45RO+ subsets. Within the CD45RO- subset further clustering is based on age and within the CD45RO+ subset based on CD4 or CD8 expression.


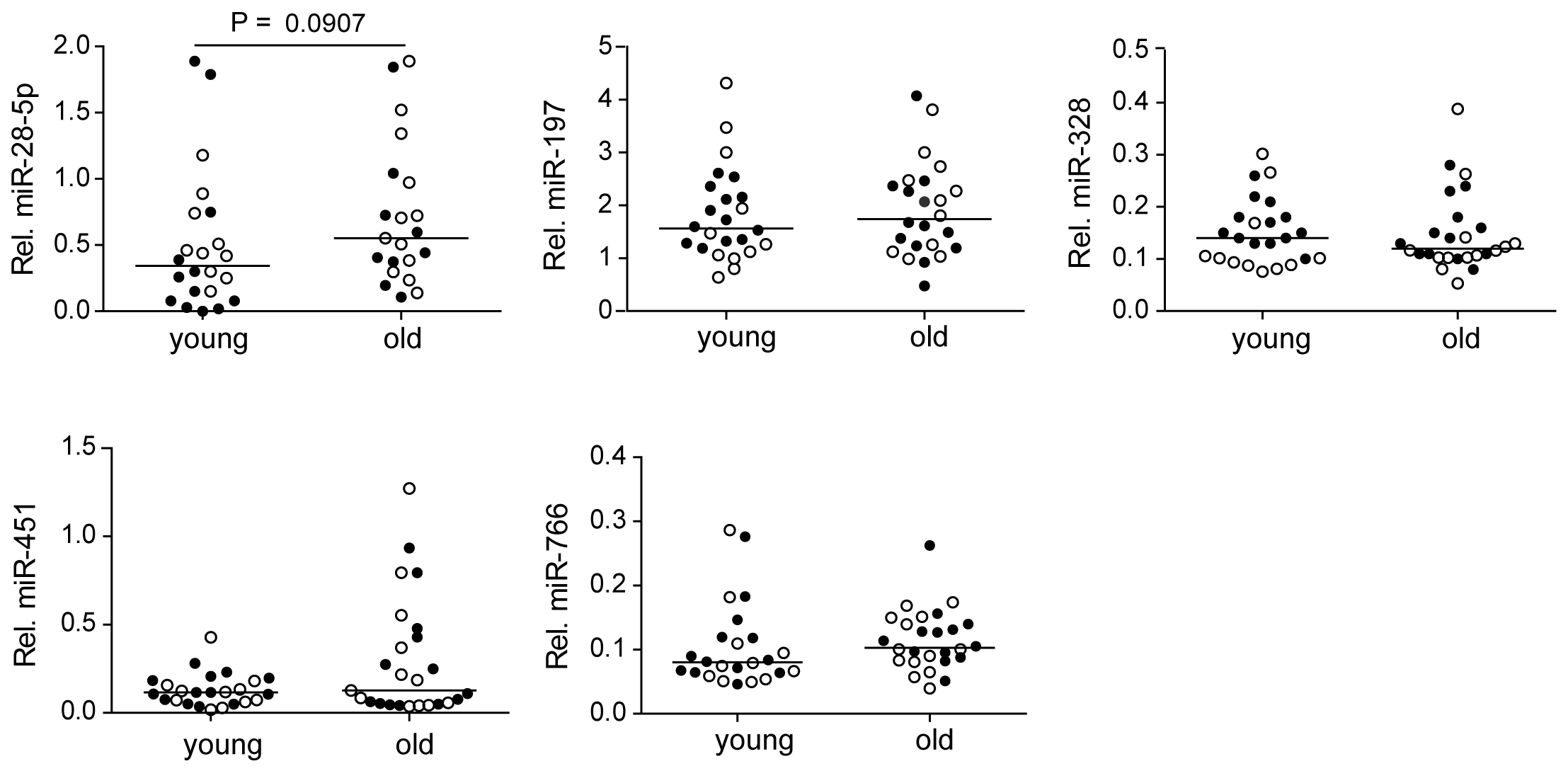


**Figure D in S1 File. Validation of microRNA array.**  Expressions of miR-197, miR-28-5p, miR-451, miR-328 and miR-766 were normalized to the expression of RNU49 (line indicates median). For miR-22, miR-483-3p, miR-885-3p and miR-574-3p the Ct values of miRNAs were above 35. For miR-574-5p the design of a custom assay failed due to high content of G-U (guanine-uracil) repeats. Filled symbols indicate CD4+ T cells and open symbols indicate CD8+ T cells.


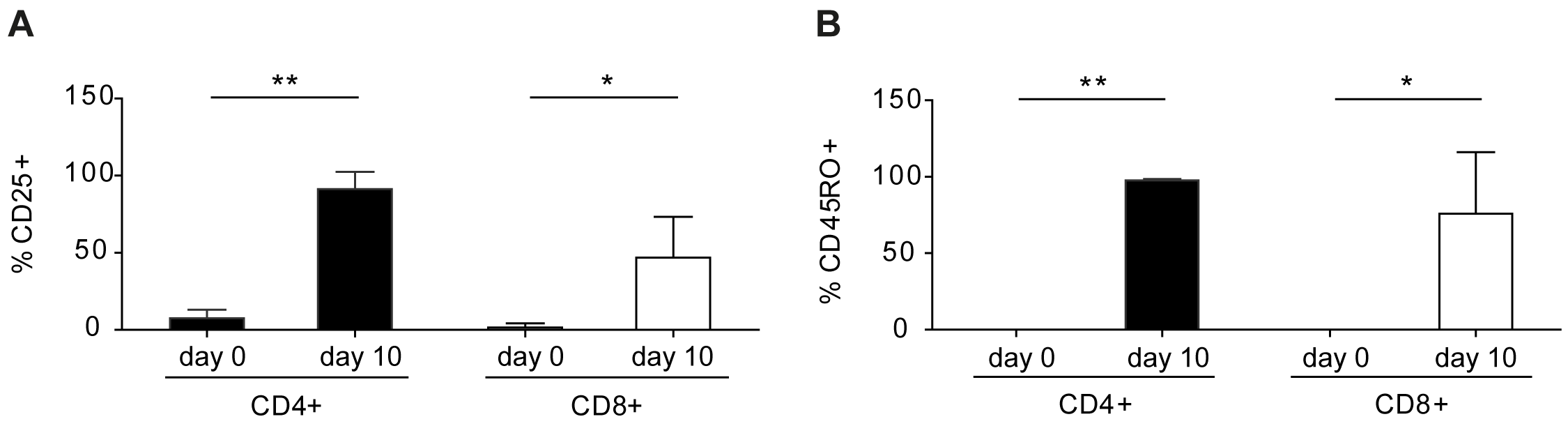


**Figure E in S1 File. Phenotype of activated CD45RO- T cells.** Expression of **(A)** cell surface activation marker CD25 and **(B)** CD45RO analyzed at day 10 following stimulation with anti-CD3/CD28, depicted as percentages (%). Black bars indicate CD4+ T cells, open bars indicate CD8+ T cells,*p ≤ 0.05, **p ≤ 0.01.
